# Brain Tumour Classification Model Based on Spatial Block–Residual Block Collaborative Architecture with Strip Pooling Feature Fusion

**DOI:** 10.3390/jimaging11120427

**Published:** 2025-11-29

**Authors:** Meilan Tang, Xinlian Zhou, Zhiyong Li

**Affiliations:** School of Computer Science and Engineering, Hunan University of Science and Technology, Xiangtan 411100, China

**Keywords:** feature fusion, spatial blocks, residual blocks, strip pooling, brain tumour MRI classification

## Abstract

Precise classification of brain tumors is crucial for early diagnosis and treatment, but obtaining tumor masks is extremely challenging, limiting the application of traditional methods. This paper proposes a brain tumor classification model based on whole-brain images, combining a spatial block–residual block cooperative architecture with striped pooling feature fusion to achieve multi-scale feature representation without requiring tumor masks. The model extracts fine-grained morphological features through three shallow VGG spatial blocks while capturing global contextual information between tumors and surrounding tissues via four deep ResNet residual blocks. Residual connections mitigate gradient vanishing. To effectively fuse multi-level features, strip pooling modules are introduced after the third spatial block and fourth residual block, enabling cross-layer feature integration—particularly optimizing representation of irregular tumor regions. The fused features undergo cross-scale concatenation, integrating both spatial perception and semantic information, and are ultimately classified via an end-to-end Softmax classifier. Experimental results demonstrate that the model achieves an accuracy of 97.29% in brain tumor image classification tasks, significantly outperforming traditional convolutional neural networks. This validates its effectiveness in achieving high-precision, multi-scale feature learning and classification without brain tumor masks, holding potential clinical application value.

## 1. Introduction

Brain tumors are the most common primary tumors of the central nervous system [1]. They form due to the uncontrolled growth of abnormal cells within the brain, severely impairing bodily functions and potentially leading to death [2,3]. Common brain tumor types include meningiomas, gliomas, and pituitary adenomas [4], each exhibiting distinct pathological characteristics and treatment approaches. Thus, timely and accurate classification and diagnosis are crucial for guiding treatment plans and improving patient prognosis. Among medical imaging technologies, magnetic resonance imaging (MRI) has become the primary tool for brain tumor detection and analysis due to its high resolution and superior soft tissue contrast [5]. MRI not only provides high-quality two-dimensional and three-dimensional images but also requires no invasive procedures, making it widely used in cancer detection and classification [6,7]. However, relying on radiologists for manual evaluation of MRI images is not only a time-consuming and labor-intensive task but may also lead to inconsistent or inaccurate diagnostic results due to human factors.

To alleviate the workload of physicians and enhance diagnostic accuracy, AI-based automated approaches have garnered significant attention in recent years [8]. Traditional machine learning methods, such as Fuzzy C-Means (FCM), Principal Component Analysis (PCA), decision trees, conditional random forests, and Support Vector Machines (SVM), have been applied to brain tumor segmentation and classification tasks [2]. However, these methods heavily rely on expert feature selection, and the quality of feature representation directly impacts model performance, making them prone to limitations and errors [9]. In contrast, deep learning approaches, particularly convolutional neural networks (CNNs), enable end-to-end automatic extraction of discriminative features from raw brain images, significantly advancing medical image analysis [10]. CNNs have achieved remarkable results in brain tumor classification, distinguishing not only benign from malignant tumors but also performing binary classification (e.g., differentiating high-grade from low-grade gliomas) and multi-class classification tasks [11,12]. Furthermore, deep learning has demonstrated outstanding performance in brain tumor segmentation, effectively predicting and detecting various lesion types [13,14]. Despite extensive research applying deep learning to brain tumor classification, key challenges persist: on one hand, models must balance the extraction of global contextual information with local details, particularly the boundaries and texture information of small tumor regions; on the other hand, existing methods exhibit insufficient equilibrium between global and local features, compromising classification accuracy and generalization capabilities.

Against this backdrop, this paper proposes a brain tumor classification method based on a spatial block–residual block collaborative architecture with strip pooling feature fusion. This approach enhances the modeling of local fine-grained features through spatial block structures, optimizes deep information propagation via residual blocks, and achieves efficient cross-region contextual information fusion using strip pooling. This integration balances global and local features, thereby improving classification accuracy and generalization capabilities. Furthermore, this approach operates independently of tumor masks. This research offers a novel technical pathway toward efficient and reliable automated brain tumor diagnosis. Ablation experiments and performance comparisons on a public dataset encompassing four tumor types validate the proposed method’s effectiveness. Key contributions include

- The model’s low-level component comprises three spatial blocks: To enhance local fine-grained features in brain tumor regions, three spatial blocks are introduced, each containing a 3 × 3 convolutional layer, batch normalization, and a ReLU activation function.
- The model’s deep-level component comprises four residual blocks: To enhance the model’s ability to extract global features while mitigating gradient vanishing, four residual blocks are introduced, each containing two 3 × 3 convolutional layers.
- Propose a novel architecture: By introducing an innovative striped pooling combination structure, the outputs from the end of the low-level spatial blocks and the end of the deep-level residual blocks are concatenated and fused. This design enhances the extraction of both global and local features, improving classification accuracy.
- Eliminate dependency on tumor masks: Our proposed model does not rely on tumor masks, enhancing its feasibility and universality in clinical practical applications.

## 2. Related Work

In brain tumor classification research, deep learning methods combined with optimization algorithms are widely applied to enhance model accuracy, sensitivity, and specificity. Optimization techniques dynamically adjust model parameters to improve the model’s fit to MRI medical imaging data, thereby strengthening its generalization capabilities. For instance, Geetha et al. [15] proposed a framework based on the Sine Cosine Archimedean Optimization Algorithm (SCAOA). This approach first employs Gaussian filtering for preprocessing and noise reduction, followed by tumor region segmentation using SegNet. Features are then extracted and fed into a shallow CNN for detection, with final classification performed by DenseNet. SCAOA is integrated to optimize the model parameters. This approach achieved 93.0% accuracy, 92.3% sensitivity, and 92.0% specificity in a three-classification task for pituitary tumors, gliomas, and meningiomas. Celik et al. [16] designed a hybrid method combining CNNs for feature extraction with classical machine learning algorithms for classification. They employed Bayesian optimization to select optimal hyperparameters while comparing nine advanced CNN models. The results show that its hybrid model achieved an average accuracy of 97.15%, with recall and precision both reaching 97%, and its classification efficiency significantly outperformed models such as InceptionV3. Another study [17] applied Bayesian Multi-Objective Optimization (BMO) to tune key parameters of the Vision Transformer (ViT) (patch_size, dim, heads, depth, mlp_dim), reducing model size by a factor of four while doubling inference speed. Validation accuracy, F_1_ Score, and precision improved by 1.48%, 3.23%, and 3.36%, respectively, highlighting BMO’s potential for efficient, lightweight brain tumor classification.

Regarding convolutional neural networks and their improvements, Rammurthy and Mahesh [18] proposed a Deep CNN based on Whale–Harris Harris Optimization (WHHO), achieving low error rates on small datasets but with limited data scalability. Islam et al. [19] designed a scheme integrating superpixels, PCA, and templated K-means (TK-means), offering low computational complexity and fast detection advantages, but exhibiting insufficient adaptability to high-dimensional clinical MRI images. Masood et al.’s [2] Mask RCNN approach utilizes segmentation masks and bounding boxes for precise localization with good robustness, yet optimization of hyperparameters failed to significantly enhance detection accuracy. The VGG-SCNet model proposed by Majib et al. [20] enables automatic classification without human intervention but lacks tumor grading capabilities. Shah et al. [21] achieved lightweight and efficient performance by fine-tuning EfficientNet-B0 combined with image augmentation, yet failed to fully validate their approach on large-scale MRI datasets. The DM-VGG16 model proposed in [22] integrates a deep Maxout network with VGG-16 through NLM filtering preprocessing, TKFCM segmentation, and multiple feature extraction methods. It achieves 90.76% accuracy, 90.65% true negative rate, and 90.75% true positive rate, highlighting the potential of deep model integration in early brain tumor detection. Togacar et al. [23] developed BrainMRNet for rapid tumor distribution detection in large datasets, though excessive residual blocks increased network complexity. Vankdothu and Hameed [24] proposed RCNN, which excelled in computational speed and complexity but demanded high storage resources. Concurrently, hybrid and ensemble approaches further drove performance improvements. Senan et al. [25] fused AlexNet with ResNet-18, combined with an SVM classifier and hypercolumn feature extraction, achieving good classification at low computational cost, though with a relatively high misclassification rate. Islam et al. [26] and Mahmud et al. [27] respectively employed ensemble learning techniques to effectively capture fine-grained features and demonstrate strong performance in multi-class detection, yet still lacked robustness for heterogeneous patient populations. The CNN architecture proposed by Abdusalomov et al. [28] demonstrated reliable performance in early detection but failed to integrate multi-source patient information. Jader et al. [29] utilized YOLOv7 to enhance detection speed and multi-class classification accuracy, yet the model exhibited shortcomings in interpretability and coverage of diverse brain injuries.

In recent years, the introduction of attention mechanisms and novel architectures has significantly driven breakthroughs in classification accuracy. Mishra et al. [30] combined CNNs with Graph Attention Autoencoders (GATE), achieving 99.83% accuracy by computing neighborhood pixel attention and feeding it into the CNN. Islam et al. [31] constructed an EfficientNet-based model incorporating preprocessing techniques like intensity normalization and image augmentation alongside custom layers, attaining a peak accuracy of 99.69%. Another study [32] proposed a multimodal approach integrating attention, convolutions, and LSTMs to directly process 3D MRI images. Combined with a weighted majority voting SVM classifier, it achieved 98.90% and 99.29% accuracy on the BRATS2015 and BRATS2018 datasets, respectively. Ullah et al. [33] introduced novel residual blocks into ResNet50 and combined gray wolf and Jaya algorithms to optimize feature extraction, achieving an average accuracy of 98% on the BraTS2020 and BraTS2021 datasets. Furthermore, the fuzzy least-squares twin SVM proposed in [34] considers membership degrees and local neighborhood information, achieving 93.45% accuracy in classification while significantly improving computational efficiency through linear equation solving. Alyami et al. [35] fused features extracted from AlexNet and VGG19, screened optimal features using the SalpSwarm algorithm, and fed them into an SVM classifier. Ultimately, 4111 features were selected from 8192, achieving 99.1% accuracy. Regarding graph neural networks and Transformer architectures, refs. [36,37] integrated CNNs with GNNs. By modeling image region dependencies and introducing graph convolutions within a 26-layer CNN, respectively, they effectively captured pixel similarity in non-Euclidean spaces, achieving accuracies of 93.68% and 95.01%. Furthermore, the SwinTransformer-based approach proposed in [38] enhances classification accuracy and inference efficiency through an improved sliding-window MHSA module and ResMLP, achieving a peak accuracy of 99.92% in brain tumor classification tasks. A brief summary of major studies on brain tumor classification and recognition is presented in Table 1.

Specifically, we previously proposed a novel dual-branch architecture [39]. The global branch employs ResNet50 with Multi-Head Self-Attention (MHSA) to capture global contextual information from the entire brain image, while the local branch utilizes VGG16 to extract fine-grained features from segmented brain tumor regions. However, brain tumor classification models based on this architecture rely on tumor masks and do not incorporate recognition of normal brain images, failing to fully leverage the respective strengths of the dual-branch network. Therefore, proposing a brain tumor classification model that does not require tumor masks and achieves high classification accuracy is crucial. The innovation of this study lies in organically integrating the strengths of these disparate architectures through a specific fusion strategy, thereby achieving optimisation for brain tumour image classification tasks. Unlike traditional multi-scale fusion approaches, our method not only performs fusion at multiple scales but also leverages the advantages of strip pooling in capturing local textural features within images, enhancing the model’s sensitivity to tumour region details. Moreover, compared to attention-based pooling approaches, our method features a more streamlined architecture with reduced computational overhead, whilst delivering consistent performance gains.

## 3. Methodology

**Figure 1:**
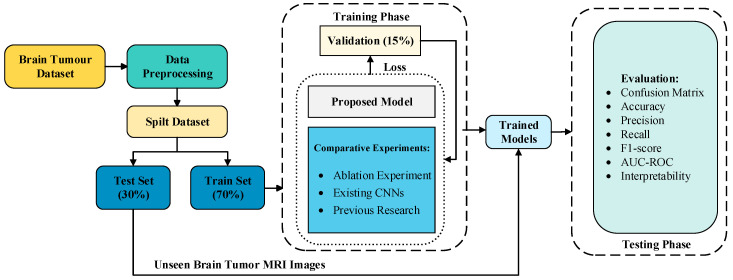
Flowchart of the Proposed MRI Classification Technique for Brain Tumours.

This paper proposes a hybrid model combining shallow stacked VGG spatial blocks with deep stacked ResNet residual blocks for the automatic classification of brain tumors in MRI images. Specifically, the model extracts low-level features of brain tumors through three shallow stacked spatial blocks, while capturing high-level features—such as the relationship between tumors and surrounding tissues and global context—via four deep stacked residual blocks. We evaluated the classification performance of our proposed method using standard metrics and validated its effectiveness by comparing it with existing convolutional neural networks (CNNs). Figure 1 illustrates the technical flowchart of the brain tumor classification technique developed in this study.

### 3.1. Architectural Components of the Brain Tumor Classification Model

To effectively fuse low-level and deep-level feature information, the proposed method introduces strip pooling structures after the third spatial block and the fourth residual block, respectively. This handles irregular tumor features and performs concatenation fusion. The fused features are concatenated and input into the Softmax classifier for the final classification task. By combining shallow and deep features, this model significantly improves brain tumor classification accuracy while avoiding reliance on tumor masks.

#### 3.1.1. Spatial Blocks and Residual Blocks

**Figure 2:**
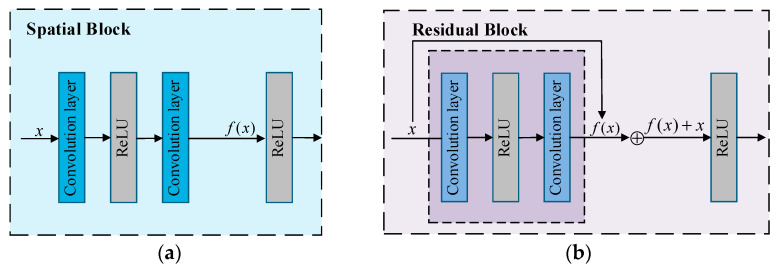
Schematic Diagram of Spatial Block and Residual Block Operations, where (**a**) is the spatial block and (**b**) is the residual block.

Spatial blocks are the fundamental building blocks within the VGG network, typically comprising a convolutional layer, batch normalization layer, activation function (ReLU), and pooling layer. At the early, low-level stages of the network, spatial blocks progressively reduce the spatial dimensions of images, aiding in capturing fundamental patterns such as edges and textures—low-level features. In brain tumor classification tasks, this process effectively extracts local, fine-grained features from tumor regions, providing richer input information for subsequent classification. As shown in Figure 2, within a spatial block, the input x sequentially passes through each operational module with corresponding operators applied, ultimately yielding the output. This process can be described by the following formula. The primary distinction between feedforward spatial blocks and residual blocks lies in the latter’s incorporation of skip connections. Residual blocks directly transmit the input x to the output of the encoding block and add this output to the original input. This process is also expressible through a formula.

The model proposed in this study comprises three spatial blocks in its lower-level section. Each spatial block contains a 3 × 3 convolutional layer, a batch normalization layer, and a ReLU activation function. The convolutional layer extracts local feature patterns associated with tumors, while the ReLU activation function enhances the model’s expressive power by introducing nonlinear characteristics. To further capture regional homogeneity and boundary-related features of brain tumors, the first two spatial blocks incorporate max pooling and average pooling operations, respectively, thereby improving the perception of features across different regions. The final spatial block employs a combined structure with striped pooling to further enhance feature extraction and classification performance.

At the deeper levels, the model consists of four residual blocks, each containing two 3 × 3 convolutional layers. The first convolutional layer is followed by a batch normalization layer and a ReLU activation function, while the second convolutional layer is followed by a ReLU activation function. A strip pooling combination structure is introduced after the final residual block to concatenate and fuse with low-level strip pooling features, thereby further enhancing the expressive power of multi-scale features. Through this structural design, the model effectively extracts fine-grained features of brain tumors and achieves information fusion across different levels, significantly improving classification accuracy.

Assuming the input feature map F has a size of M×N, the convolution kernel *k* has a size of r×s, $k_{p,q}$ denotes the weight of the convolution kernel at position $\left(p,q\right)$, $f_{m,n}$ represents the value of the input feature at position $\left(m,n\right)$, and the output feature map Y has a size of (M−r+1)×(N−s+1), then the output $y_{m,n}$ of the convolution operation can be expressed as the weighted sum of the corresponding local region of the input feature map, as shown in Formula (1).

(1) $$y_{m,n}=\sum_{p=1}^{r}\sum_{q=1}^{s}f_{m+p-1,n+q-1}\cdot k_{p,q}$$

Assuming the size of the pooling kernel is r×s and the stride is t, average pooling and max pooling can be expressed by Formula (2) and Formula (3), respectively.

(2) $$y_{m,n}^{avg}=\frac{1}{r\times s}\sum_{p=1}^{r}\sum_{q=1}^{s}f_{m+i-1,n+j-1}$$

(3) $$y_{m,n}^{max}=\underset{1\leq i\leq r,1\leq j\leq s}{\max}f_{m+i-1,n+j-1}$$

#### 3.1.2. Strip Pooling

Strip Pooling [40] is an improved pooling method designed to more effectively handle irregularly shaped objects and enhance the representational power of image features. Traditional pooling methods such as max pooling or average pooling typically use square windows, such as 2 × 2 or 3 × 3, that slide across the image to aggregate information from local regions. These methods extract useful information through dimensionality reduction and feature aggregation while reducing computational complexity. However, traditional pooling methods implicitly assume objects in images have regular shapes, making square windows effective for regular shapes. In brain tumor classification tasks, tumors typically exhibit irregular morphologies with complex boundaries and structures. The square windows used in traditional pooling methods may introduce substantial irrelevant background information when processing such irregular shapes, leading to insufficient feature extraction accuracy or noise introduction. To address this issue, this study introduces a combined structure comprising striped pooling, average pooling, Dropout, and fully connected layers at the end of spatial blocks in the lower layers of the network and at the end of residual blocks in the deeper layers. By fusing their features, this approach enhances the model’s representational capability.

Strip pooling captures irregularly shaped features in brain tumor images more effectively by performing pooling operations vertically and horizontally. Horizontal strip pooling performs pooling operations along the row direction (i.e., horizontally) of the image. Given an input feature map *Z*∈ ℝ*^H^*^×*W*×*C*^, where *H* denotes height, *W* denotes width, and *C* denotes the number of channels, horizontal strip pooling aggregates across the width while fixing the height, thereby extracting global structural information across channels. The operation of horizontal strip pooling is expressed by Equation (4). Here, $O_{h,i,c}^{h}$ denotes the value of the output feature map at position $\left(h,i,c\right)$, where *h* is the row index, *i* is the column index, *c* is the channel index, and *W* represents the width of the feature map. This equation indicates that for each column h and each channel c, the average is computed along the width W to obtain the pooling result.

(4) $$O_{h,i,c}^{h}=\frac{1}{W}\sum_{j=1}^{W}F_{h,j,c}$$

Correspondingly, vertical strip pooling performs pooling operations along the column direction (i.e., vertically) of the image, as expressed by Formula (5). Here, $O_{j,i,c}^{v}$ denotes the value of the output feature map at position $\left(j,i,c\right)$, where *j* is the column index, *i* is the row index, *c* is the channel index, and *H* represents the height of the feature map. This formula indicates that for each column j and each channel *c*, the average is computed along the height *H* to obtain the pooling result.

(5) $$O_{j,i,c}^{v}=\frac{1}{H}\sum_{h=1}^{H}F_{h,j,c}$$

**Figure 3:**
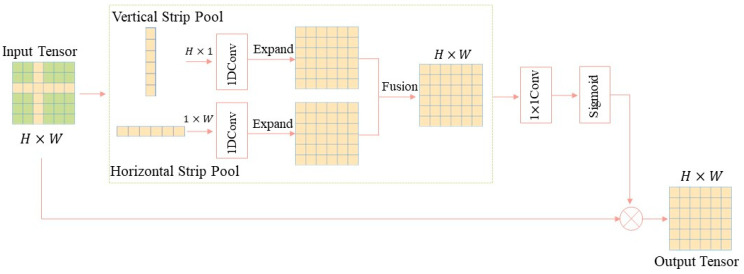
Schematic Diagram of the Strip Pooling Process.

As shown in Figure 3, strip pooling operations are first applied horizontally and vertically to the input feature map to obtain pooling feature maps in both directions. Subsequently, these two types of pooling feature maps are separately fed into a 1D convolutional layer with a kernel size of 3 to generate new feature representations. The feature maps are then normalized and expanded to match the dimensions of the input feature map. The expanded feature maps fuse the pooling features from both directions through element-wise summation. The fused feature map then undergoes a 1 × 1 convolution layer, generating weight matrices for the height and width directions via a Sigmoid activation function. Finally, these weight matrices are multiplied element-wise with the original feature map to enhance attention in the height and width directions, yielding the weighted feature representation. Traditional square pooling assumes locally regular structures and often averages away fine boundary details. In contrast, strip pooling performs one-dimensional pooling along horizontal and vertical directions, which captures elongated or anisotropic edge features more effectively while reducing background interference. This mechanism expands the receptive field in one direction without increasing parameters, thus better preserving irregular lesion boundaries. This interpretation aligns with the irregular edge modelling research in [41], which demonstrates that the aggregation of anisotropic features can enhance the characterisation of geometric edges.

### 3.2. Overall Model Architecture

The model proposed in this study aims to leverage the powerful feature learning capabilities of convolutional neural networks (CNNs) to automatically extract tumor feature patterns from brain MRI scans. The proposed model relies solely on whole-brain images as input, without requiring tumor mask files. Its overall architecture draws inspiration from both the VGG network and ResNet, combining their strengths to enhance feature extraction capabilities. In the shallow layers, the model incorporates the spatial block structure commonly used in VGG networks to extract fine-grained local features. It further enhances regional uniformity and boundary information extraction by combining convolutional operators with max pooling and average pooling operations. In the deep layers, the model introduces ResNet’s residual block structure to capture relationships between tumors and surrounding tissues, as well as deeper global contextual features.

**Figure 4:**
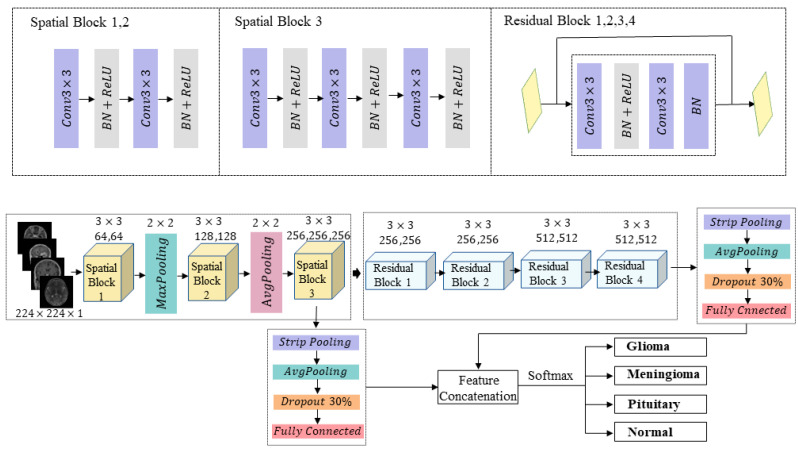
Detailed components of the model and its overall structure.

To further enhance feature expression capabilities, this model introduces a novel strip pooling combination structure at both the low-level spatial blocks and the end of deep residual blocks, subsequently concatenating and fusing the two. This structure comprises strip pooling, average pooling, Dropout, and a fully connected layer. The strip pooling effectively captures spatial information from different directions, thereby strengthening the model’s adaptability to tumor morphological diversity and irregularities. Finally, the model concatenates the two feature sets processed by the strip pooling combination structure along the channel dimension. The fused features are then fed into a Softmax classifier to complete the final tumor classification task. By integrating multi-scale feature information from both shallow and deep layers, this model not only effectively extracts key image features and improves classification accuracy but also mitigates overfitting to a certain extent. Figure 4 illustrates the overall architecture of the proposed model and details of its constituent modules.

### 3.3. Model Hyperparameter Settings

The brain tumor dataset used in this study was split into training and validation sets at a 7:3 ratio. Within the training set, an additional 15% was allocated as an internal validation set for cross-validation during parameter tuning. Model hyperparameters—including learning rate, batch size, and number of iterations—were optimized using cross-validation techniques. During training, AdamW [42] was selected as the optimization algorithm after evaluating multiple options. Compared to Adam, AdamW retains its advantages while achieving more effective weight decay, thereby enhancing model stability and training efficiency. This choice was based on performance evaluations of different optimizers for the task at hand. During model optimization, the Softmax activation function was employed, and training utilized the cross-entropy loss function. To obtain the optimal model, a checkpoint mechanism was implemented to save model parameters when validation accuracy improved. Additionally, loss curves were recorded during training and validation to observe how model performance evolved with training iterations. The optimal hyperparameter configuration is shown in Table 2.

## 4. Experiments and Analysis

All end-to-end experiments based on deep CNNs and data preprocessing steps were implemented using MATLAB R2021b on a computer equipped with an Intel^®^ Core™ i5-14600KF CPU, an NVIDIA GeForce RTX 5060 GPU and 16GB RAM. Performance analysis plots for some experiments were generated using the Python (version 3.1.2) based matplotlib library within PyCharm (version 2025.1.1) software. The proposed model contains approximately 9.8 million trainable parameters.

### 4.1. Datasets

**Figure 5:**
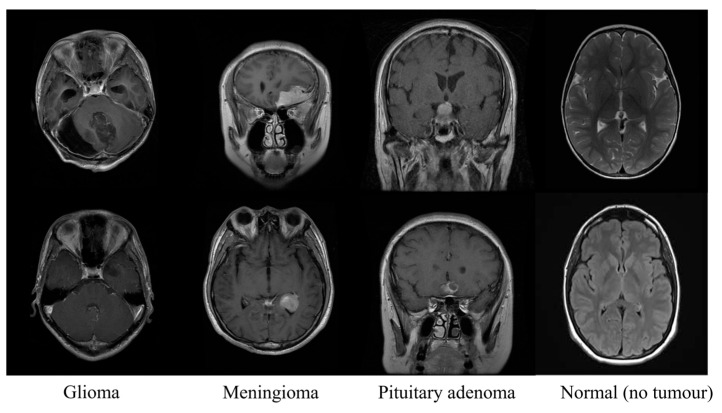
Example images for each tumour type.

The dataset used in this paper is the publicly accessible Kaggle “Brain Tumor MRI Dataset,” which does not include tumor mask files. It is combined with datasets from three additional sources: the Figshare dataset [43], the SARTAJ dataset, and the Br35H dataset [44]. The total dataset comprises 7023 human brain MRI scans, including 1621 glioma images, 1645 meningioma images, 1757 pituitary tumor images, and 2000 non-tumor images. To ensure transparency and reproducibility in the utilisation of multimodal data, we adhered to the rigorous multimodal fusion and subject-level segmentation protocol proposed in [45]. Simultaneously, we generate a globally unique ID for each image while retaining the original dataset ID, managing these via a mapping table. Potential duplicate samples are identified by matching patient IDs to prevent double counting. Following deduplication, a unified splitting strategy is applied to partition the data into training, validation, and test sets based on the globally unique ID. This ensures images from different dataset sources do not appear across multiple sets, thereby preventing data leakage. The dataset is split into training and testing sets at a 7:3 ratio, as detailed in Table 3. Figure 5 displays representative images from each of the four tumor categories within the dataset. In addition, we cross-checked the metadata and image identifiers from each dataset to confirm that there is no duplication or overlap among the sources.

### 4.2. Data Preprocessing

All brain tumor MRI images underwent a series of preprocessing steps to enhance the effectiveness of feature extraction and the overall model performance. While these steps—including image resizing, RGB-to-grayscale conversion, denoising via an unsharp mask, and edge enhancement using a Sobel filter [46]—are fundamental, they collectively serve to standardize the input and accentuate critical tumor characteristics. Specifically, each MRI image was first resized to a uniform dimension of 224 × 224 × 3. It was then converted to a grayscale image (224 × 224 × 1) to reduce computational complexity. Subsequently, a denoising operation was performed using a 100-pixel radius unsharp mask to improve image quality by reducing noise. Finally, a Sobel filter was applied to sharpen and highlight the edge information of the tumor regions. Although the visual differences after each intermediate step are subtle, this streamlined preprocessing pipeline is crucial for enhancing image quality, minimizing noise interference, and emphasizing the discriminative features of tumor regions, thereby providing cleaner and more reliable input for the subsequent classification model. It is worth noting that the preprocessing steps applied in this study are fundamental and have a limited impact on the overall classification performance. Therefore, the ablation study focuses primarily on the architectural components of the proposed model. Additionally, during the training phase, we applied conventional data augmentation techniques including random rotation (±15°), horizontal flipping, slight translation (≤10%), and brightness/contrast adjustments (±10%). The optimal range for these parameters was determined through tuning on the validation set. For regularisation, we employed dropout (at a rate of 0.3) and L2 weight decay (with a coefficient of 1 × 10^−4^) to further enhance model stability.

### 4.3. Evaluation Indicators

In classification tasks, evaluating performance metrics is crucial because different models may prioritize optimizing distinct evaluation criteria. To comprehensively assess the performance of brain tumor classification models, this study employs widely used evaluation metrics, including accuracy, precision, recall, and F_1_ score. These metrics are calculated based on the confusion matrix, which consists of four fundamental elements: true positives (*TP*), true negatives (*TN*), false positives (*FP*), and false negatives (*FN*). Through these elements, various performance metrics can be further computed to comprehensively evaluate the model’s performance across different classification tasks.

Accuracy serves as a key metric for evaluating the correctness of a classification model. As shown in Formula (6), Accuracy is calculated as the ratio of correctly predicted samples to the total number of samples.

(6) $$\mathrm{Accuracy}=\frac{TP+TN}{TP+FN+FP+TN}$$

Precision and Recall are commonly used metrics for evaluating classifier performance. In a three-class classification task, precision is defined as the proportion of samples actually belonging to a certain class among those predicted by the classifier to belong to that class, as shown in Formula (7). Recall, on the other hand, refers to the proportion of samples that actually belong to a certain class that are correctly predicted by the classifier, as shown in Equation (8).

(7) $$\mathrm{Precision}=\frac{TP}{TP+FP}$$

(8) $$\mathrm{Recall}=\frac{TP}{TP+FN}$$

The F_1_ Score is a comprehensive metric that considers both precision and recall, providing a holistic measure of a classification model’s performance. The formula for calculating the F_1_ Score is given in (9).

(9) $$F_{1}\;\mathrm{Score}=2\frac{\mathrm{Precision}\times \mathrm{Recall}}{\mathrm{Precision}+\mathrm{Recall}}$$

### 4.4. Performance Comparison of Different Optimizers

This paper conducts experiments comparing the performance of different optimizers to select the most suitable one for this model. Specifically, four commonly used optimizers were employed, each configured with 50 iterations. Learning rates and batch sizes were adjusted based on literature recommendations: learning rates were set to 0.001, 0.0001, and 0.00001, while batch sizes were set to 16, 32, and 64 for model training. Ultimately, the optimal optimizer was selected by comparing each optimizer’s performance under its best hyperparameter configuration. As shown in Table 4, this study compared the validation set performance of different optimizers under their optimal hyperparameters, calculating precision, recall, and F_1_ score using macro-average. Results indicate significant performance variations across optimizers for the proposed model. Specifically, the model achieved its best performance on the validation set when using the AdamW optimizer with a learning rate of 0.0001 and a batch size of 32, attaining an accuracy of 97.29%. Although other optimizers also demonstrated good performance under their optimal hyperparameters, they remained significantly below AdamW. Therefore, AdamW was ultimately selected as the optimizer for training the model in this paper.

### 4.5. Ablation Studies

A series of ablation experiments systematically analyzed the core components of the model and compared their classification accuracy, with results shown in Table 5. Here, Strip Pooling 1 and Strip Pooling 2 represent the strip pooling combination structures applied to the shallow and deep layers of the network, respectively. Specifically, Model A contains only spatial blocks; Model B introduces a strip pooling combination structure at the end of spatial blocks; Model C consists of residual blocks; Model D adds a strip pooling structure after residual blocks; Model E stacks spatial blocks and residual blocks; Model F represents the complete model proposed in this chapter. Comparing Models A and B, as well as Models C and D, reveals that incorporating strip pooling structures into both spatial blocks and residual blocks improves classification accuracy by 1.18% and 0.53%, respectively. Further comparison between Models E and A/C shows that using both spatial blocks and residual blocks in shallow and deep layers, respectively, yields higher accuracy than relying solely on either spatial blocks or residual blocks. Moreover, compared to Model E, the proposed Model F achieves a 2.18% increase in classification accuracy by simultaneously introducing strip pooling structures in both the lower-layer spatial blocks and upper-layer residual blocks while integrating feature information. These experimental results validate the rationality and effectiveness of the designed model.

### 4.6. Performance Comparison with Baseline Methods

**Figure 6:**
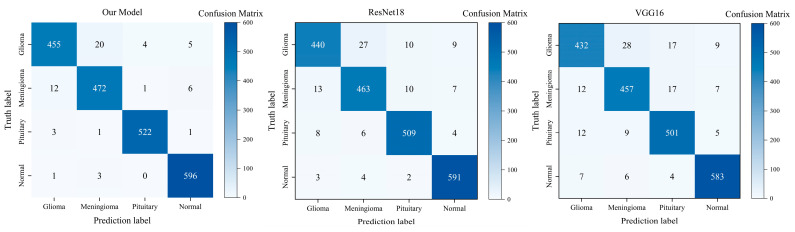
Confusion matrices of the proposed model and two baseline models.

To validate the effectiveness of the proposed method, this section evaluates it against the ResNet18 model based on residual learning and the VGG16 model based on spatial blocks under identical experimental conditions. These two models were selected as baselines due to their network depth being comparable to the proposed model. In addition, transfer learning strategies were employed across all models to accelerate the convergence process and enhance classification performance. Specifically, VGG16 achieves downsampling through convolutional layers and max-pooling layers, while ResNet18 employs strided convolutions to replace pooling operations. The proposed method in this chapter utilizes pooling operations in shallow layers and strided convolutions in deep layers. It combines striped pooling to process shallow and deep feature information separately, followed by feature fusion and classification to enhance overall performance. Experimental results are presented in Table 6, listing precision, recall, F_1_ score, and overall accuracy for each category. Results indicate that the residual-block-based ResNet18 model achieves an overall classification accuracy of 95.11%, while the spatial-block-based VGG16 model reaches 93.68%. In contrast, the proposed model outperforms both architectures across all performance metrics. All metrics were computed from confusion matrices, with the three models’ matrices shown in Figure 6. This demonstrates that the proposed model significantly surpasses the VGG16 and ResNet18 baseline models in recognition performance across all categories.

### 4.7. Performance Comparison with Existing Convolutional Neural Network Models

To further validate the performance of the proposed model, this study compares it with several classical convolutional neural network (CNN) classification models, including Inception-V3, Xception, GoogleNet, MobileNet-V3, and DenseNet121. To adapt to the brain tumor classification task, fully connected layers and classification layers were added to each baseline network, and training was conducted in an end-to-end manner. To ensure fair comparison, all baseline models were trained and validated using identical dataset partitioning schemes and preprocessing workflows. All models were implemented via transfer learning to accelerate convergence and enhance generalisation capabilities. Table 7 presents the performance comparison results across models in terms of accuracy, precision, recall, and F_1_ score under identical experimental conditions. Results demonstrate that the proposed model outperforms other classical CNN models across all performance metrics. This advantage primarily stems from its systematic incorporation of max-pooling and average-pooling in spatial blocks, residual block architecture, and strip pooling to fuse shallow and deep feature information, thereby significantly enhancing overall classification performance. Notably, DenseNet121 demonstrated relatively superior performance with a classification accuracy of 95.30%. This result may be attributed to its deeper network architecture, which enables extraction of richer high-level features. However, this model exhibits significantly higher computational complexity and runtime compared to the proposed method. In contrast, Xception showed relatively lower performance with an accuracy of only 89.36%.

### 4.8. Analysis of Model Performance

**Figure 7:**
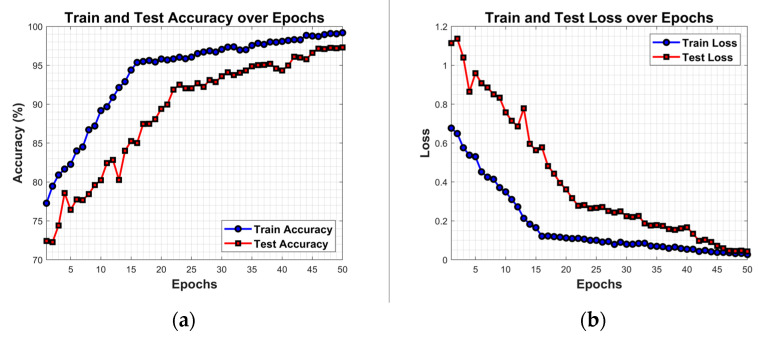
Accuracy and loss curves of the model on the training and test sets, with the accuracy curves on the left in (**a**) and the loss curves on the right in (**b**).

To evaluate the model’s training process and generalization capability, accuracy curves and loss curves for the training and test sets were plotted, as shown in Figure 7. The results indicate that during training, the model’s accuracy progressively improved while the loss value continuously decreased, demonstrating a favorable convergence trend. Although the test set accuracy and loss exhibited some fluctuations in the early training stages, they overall showed a gradual stabilization trend. As training progressed, the test set accuracy steadily increased while the loss gradually decreased. By the 50^th^ epoch, the test set curve converged with the training set curve, indicating that the model effectively mitigated overfitting risks and demonstrated strong generalization capabilities.

**Figure 8:**
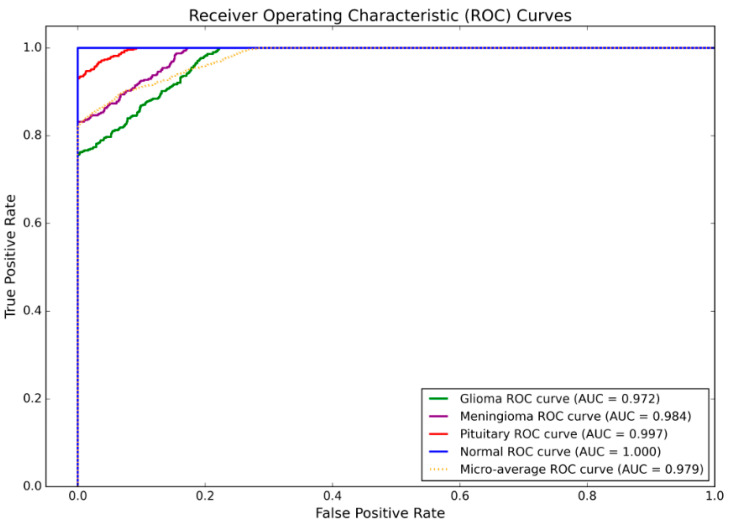
ROC Curve for Each Category.

To further evaluate the performance of the proposed model in brain tumor classification tasks, ROC curves were plotted for each category, and a Micro-average ROC curve was calculated to assess overall classification performance, as shown in Figure 8. The results indicate that the model exhibits varying levels of discrimination across different categories. Specifically, the AUC values for glioma, meningioma, pituitary tumor, and normal categories reached 0.972, 0.984, 0.997, and 1.000, respectively, while the Micro-average AUC value was 0.979. The model demonstrated optimal discrimination performance in the normal category with an AUC of 1.000, indicating its ability to accurately distinguish normal samples from pathological samples. Among tumor categories, both glioma and meningioma achieved AUC values exceeding 0.97, demonstrating strong discriminatory capability. For pituitary tumor identification, the model attained a near-perfect AUC value of 0.997, This further validates its high sensitivity and stability for this type of tumor. The results demonstrate that the proposed model exhibits excellent discriminative power and generalization capability in multi-class classification tasks, enabling it to robustly adapt to the identification requirements of different categories of brain tumors. It is worth noting that the proposed model must also account for network compression and runtime optimisation [47], employing lightweight convolutional structures to minimise computational overhead. Future work will prioritise analysis of the model’s resource consumption.

### 4.9. Performance Comparison with Other Studies

The proposed model in this study was evaluated against existing methods for brain tumor classification, with results summarized in Table 8. Previous research demonstrated that replacing convolutional neural networks with capsule networks, combined with feature attention modules and dynamic routing algorithms, achieved a classification accuracy of 94.8% and an F_1_ score of 0.933 [48]. Senan et al. [25] employed AlexNet and ResNet18 for feature extraction, combined with SoftMax and SVM classifiers. The AlexNet + SVM combination demonstrated the best performance, achieving an accuracy of 95.10% and an F_1_ score of 0.9685. Latif et al. [49] proposed a multi-class glioma classification method integrating CNN feature extraction with an SVM classifier, achieving an accuracy of 96.19%. Chitnis et al. [50] developed the LeaSE framework, which automatically searches for high-performance neural network architectures and generates high-fidelity explanations to optimize classification performance, achieving an accuracy of 90.61%. Another study proposed a lightweight residual multi-scale CNN model that selectively learns discriminative features using a lightweight global attention module (LGAM), achieving an accuracy of 96.64% and an F_1_ score of 0.9687 [51]. Although the aforementioned methods demonstrated commendable performance, their accuracy and F_1_ scores remain slightly lower than those achieved by the method proposed in this study.

## 5. Conclusions

This study proposes a brain tumor classification model that integrates low-level and high-level features to enhance the discriminative capability of complex medical images. The model architecture comprises three shallow spatial blocks and four deep residual blocks, with strip pooling modules introduced in the final spatial block and residual block, respectively. Strip pooling effectively captures irregular morphological features within tumor regions. Its output is concatenated with corresponding layer features to achieve collaborative modeling across multiple feature levels. This design fully leverages the fine-grained information of low-level features and the semantic information of high-level features, enhancing the model’s accuracy and robustness in characterizing complex lesions. During feature learning, the multi-scale feature fusion strategy effectively mitigates feature information loss and overfitting issues commonly encountered in traditional convolutional neural networks for brain image analysis. Systematic experiments and ablation studies on glioma, meningioma, pituitary tumor, and normal brain MRI datasets demonstrate that this method significantly outperforms multiple classical CNN baseline models in classification performance, ultimately achieving a classification accuracy of 97.29%. This outcome validates the proposed model’s effectiveness and application potential in intelligent brain tumor image diagnosis. Nevertheless, this study still has certain limitations, including mild class imbalance across datasets, variability in MRI acquisition protocols, and the absence of interpretability analyses such as heat map or feature activation visualizations. In future work, we plan to incorporate these visualization techniques and further optimize the model’s real-time feasibility to strengthen its clinical applicability.

## Figures and Tables

**Table 1:** Summary of Related Studies on Brain Tumor Recognition and Classification.

Reference	Model/Approach	Dataset	Accuracy (%)
[2]	Mask-RCNN	Kaggle and Figshare	98.34%
[15]	SCAOA	BRATS 2020 and Figshare	93%
[21]	EfficientNet-B0	Brats2015	98.87%
[23]	BrainMRNet	Figshare	96.73%
[25]	AlexNet + SVM	Figshare	95.10%
[28]	YOLOv7	Kaggle	99.5%
[30]	GATE-CNN	——	98.27%
[33]	ResNet-50	BraTS2021	98%

**Table 2:** Summary of Optimised Hyperparameter Settings.

Hyperparameter	Value
Batch Size	32
Learning Rate	0.0001
Optimizer	AdamW
Loss Function	Cross-Entropy
Epochs	50
Activation Functions	ReLU, Softmax
Dropout Rate	0.3

**Table 3:** Number of Training and Test Samples per Class in the Dataset.

Tumour Type	Training Set (70%)	Test Set (30%)	Total (100%)
Meningioma	1135	486	1621
Glioma	1152	493	1645
Pituitary	1230	527	1757
Normal	1400	600	2000
Total	4917	2106	7023

**Table 4:** Performance Comparison of Different Optimizers with Optimal Hyperparameter Configurations.

Optimizer	Learning Rate	Batch Size	Accuracy	Precision	Recall	F_1_ Score	Loss
AdamW	0.0001	32	97.29%	0.9721	0.9713	0.9716	0.043
Adam	0.0001	16	96.11%	0.9592	0.9587	0.9588	0.078
RMSprop	0.001	32	94.63%	0.9445	0.9437	0.9439	0.226
SGD	0.0001	64	94.97%	0.9479	0.9469	0.9472	0.131

**Table 5:** Ablation experiments on different architectures.

Model	Spatial Block 1, 2, 3	Strip Pooling 1	Residual Blocks 1, 2, 3, 4	Strip Pooling 2	Accuracy
A	✓				90.36%
B	✓	✓			91.54%
C			✓		93.39%
D			✓	✓	93.92%
E	✓		✓		95.11%
F(Ours)	✓	✓	✓	✓	97.29%

✓: Module included.

**Table 6:** Performance Comparison of Developed Models with Baseline Architecture Using Test Set.

Model	Category	Precision	Recall	F_1_ Score	Overall Accuracy
VGG16	Glioma	0.9330	0.8889	0.9104	93.65%
	Meningioma	0.9140	0.9270	0.9204	
	Pituitary adenoma	0.9295	0.9507	0.9400	
	Normal	0.9652	0.9717	0.9684	
ResNet18	Glioma	0.9483	0.9053	0.9263	95.11%
	Meningioma	0.9260	0.9391	0.9325	
	Pituitary adenoma	0.9586	0.9658	0.9622	
	Normal	0.9673	0.9850	0.9761	
Our Model	Glioma	0.9660	0.9401	0.9529	97.29%
	Meningioma	0.9516	0.9613	0.9564	
	Pituitary adenoma	0.9905	0.9905	0.9905	
	Normal	0.9803	0.9933	0.9868	

**Table 7:** Performance Comparison of Standard Classical CNNs and the Proposed Model on Test Data.

Model	Accuracy (95% CI)	Precision (95% CI)	Recall (95% CI)	F_1_ Score (95% CI)
Inception-V3	91.55% (89.03–93.43%)	0.9115 (0.905–0.923)	0.9112 (0.903–0.919)	0.9112 (0.903–0.919)
Xception	89.36% (87.67–90.91%)	0.8893 (0.881–0.898)	0.8889 (0.878–0.898)	0.8889 (0.879–0.899)
GoogleNet	91.07% (90.46–91.64%)	0.9073 (0.900–0.914)	0.9062 (0.899–0.913)	0.9064 (0.899–0.913)
MobileNet-V3	92.35% (91.73–92.89%)	0.9200 (0.913–0.929)	0.9199 (0.914–0.927)	0.9199 (0.914–0.926)
DenseNet121	95.30% (94.77–95.72%)	0.9520 (0.949–0.953)	0.9517 (0.947–0.956)	0.9518 (0.946–0.956)
Our Model	97.29% (96.96–97.60%)	0.9721 (0.969–0.974)	0.9713 (0.970–0.973)	0.9716 (0.966–0.973)

**Table 8:** Performance Comparison of the Method with Published Literature.

Model	Literature	Category Count	Accuracy	F_1_ Score
FAU-CapsNet	[48]	3	94.80%	0.9330
AlexNet + SVM	[25]	4	95.10%	0.9685
CNN + SVM	[49]	4	96.19%	0.8700
LeaSE	[50]	4	90.61%	0.9148
ARM-Net	[51]	3	96.64%	0.9687
Ours	—	4	97.29%	0.9716

## Data Availability

The original contributions presented in this study are included in the article. Further inquiries can be directed to the corresponding author.
